# Associations between match participation, maturation, physical fitness, and hormonal levels in elite male soccer player U15: a prospective study with observational cohort

**DOI:** 10.1186/s12887-022-03257-7

**Published:** 2022-04-11

**Authors:** Ebrahim Eskandarifard, Hadi Nobari, Filipe Manuel Clemente, Rui Silva, Ana Filipa Silva, Antonio José Figueiredo

**Affiliations:** 1 Department of Exercise Physiology, Faculty of Sport Sciences, University of Isfahan, Isfahan, 81746-7344 Iran; 2 Faculty of Sport Sciences and Physical Education, University of Coimbra, Coimbra, Portugal; 3 Department of Exercise Physiology, Faculty of Educational Sciences and Psychology, University of Mohaghegh Ardabili, Ardabil, 56199-11367 Iran; 4 Sports Scientist, Sepahan Football Club, Isfahan, 81887-78473 Iran; 5 Department of Physiology, Faculty of Sport Sciences, University of Extremadura, Cáceres, 10003 Spain; 6 Escola Superior Desporto e Lazer, Instituto Politécnico de Viana do Castelo, Rua Escola Industrial e Comercial de Nun’Álvares, Viana do Castelo, 4900-347 Portugal; 7 Instituto de Telecomunicações, Delegação da Covilhã, Lisboa, 1049-001 Portugal; 8 Research Center in Sports Performance, Recreation, Innovation and Technology (SPRINT), Melgaço, 4960-320 Portugal; 9 The Research Centre in Sports Sciences, Health Sciences and Human Development (CIDESD), Vila Real, 5001-801 Portugal

**Keywords:** Skeletal age, Young, Performance, Playing time, Football, Talent development

## Abstract

**Objectives:**

The aims of this study were to analyze the relationships between minutes of play (MP) and maturity status, fitness, and hormonal levels and to explain how those measures influence the time of play.

**Methods:**

Twenty-six youth soccer players U15 participated in this study over a full-season period. Anthropometric measures, maturity status, growth hormone (GH), insulin-like growth factor and physical levels such as maximal oxygen uptake (VO_2max_), fatigue index, countermovement jump (CMJ) performance were collected. At the end-season, players were assessed in 6 different tests over four days.

**Results:**

VO_2max_ largely correlated with GH (*r* = 0.57) and CMJ (*r* = 0.51). Also, GH largely correlated with CMJ (*r* = 0.55). MP had moderate correlations with VO_2max_ (*r* = 0.44) and CMJ (*r* = 0.42). Multiple linear regression with maturation, physical fitness and hormonal levels explained R^2^ of 0.62 of the MP (*F* (8, 17) = 3.47, *p* = 0.015). Although each independent variable alone was not able to determine the playing time, when using the interactions, the model significantly explained the MP.

**Conclusions:**

The combination of maturity status, physical fitness, and hormonal levels seem to play a determinant role in explaining the match participation in youth soccer players.

## Introduction

Youth soccer levels are organized by chronological age, disregarding the impact that biological age may have on the differentiation between athletes, as well as on talent selection [1]. By grouping young athletes according to their chronological age (date of birth) leads to a misconception regarding athlete’s biological age (state of maturation), which disrupts talent identification and development, mainly associated with contempt for later maturing athletes [2, 3]. Shifting these practices is of paramount, and can be possible with simple methods of monitoring maturing state of athletes, mainly through anthropometric measures to predict maturity offset, years from peak height velocity (PHV) [4, 5]. Also, the maturation level can be calculated by skeletal age estimation through the Fels method [6]. Essentially, knowing the chronological age (date of birth) and measuring the weight, standing height, sitting height, and leg length of the young athletes it is possible to predict the distance they are from PHV, through a valid and reliable calculation [4]. As the level of maturation may differ substantially among children of the same chronological age [7, 8], it is expected that within the same team exists great variability of maturational levels between young soccer players [9, 10]. In fact, in a study conducted on 159 male players aged 11–14 years old, it was revealed that within the same age group the differences in maturation was meaningful, where early mature players were morphologically advanced in relation to normal and later mature male players [11].

Moreover, it is well known that early maturing players present better physical qualities in relation to their late maturing counterparts [12–14]. For instance, a study conducted on 69 young soccer players revealed that early maturing players outperformed their late maturing colleagues in aerobic capacity, speed, strength and power variables [12]. Notwithstanding, it seems that players that are advanced in maturity concurrent with hormonal levels, presents higher technical soccer performance in relation to their late maturing counterparts [15]. Also, it has been documented that elite youth soccer training leads to greater physical qualities improvements, concomitant with growth spurt [16–18].

These improvements can also be related with hormonal changes that occur during growth, mainly associated with the actions of growth hormone (GH) and insulin-like growth factor (IGF1) which may be regulated by training stimuli [19]. The GH-IGF1 axis is responsible for the anabolic environment that results in a normal and healthy growth of children [20]. Also, at the onset and until the end of puberty it is expected an increase in this anabolic environment [21], as well as training-related effects on the production or inhibition of growth hormones [17, 22]. Further, it was documented that maturity status and hormonal activity seem to have a relationship with physical performance in soccer [17]. Although IGF1 concentration decreases due to the inflammatory responses of acute loads [23], a chronic exposure to exercise cause positive adaptations which is related to augmented circulating growth hormones [21].

As the early maturing players are normally stronger an taller than their late maturing peers [9], it is a common practice that clubs excludes late maturing players, given their smaller structure [3, 5]. As a consequence, talent identification follows an unidimensional approach where it reigns the physical-based selection, in which early maturing players are naturally more benefited [24, 25]. In fact, in a short-term perspective, some studies showed that early maturing players covered greater distances than late maturing players [13, 26]. Indeed, Goto et al. [26], revealed that in an U14 soccer team, early maturers covered greater match distances than late maturers, mainly in high intensity running and spent more time in this intensity zone [26]. These evidences may be related to the fact that on those particular competitive levels, teams are in a critical stage of maturity where hormonal changes begin to take shape in a more accentuated fashion [20, 21]. However, there is still a lack of consensus regarding those findings [27].

Coaches are expected to have a preference for selecting early maturing players for competition due to their greater morphological and physical characteristics, as well as their match performance mainly in the pubertal critical phase [3, 28]. Although these preferences exist, few evidences support the determinant dimensions influencing the minutes played by young soccer players. In fact, Goto et al. [26] revealed that more mature players have greater time of play in lower chronological age categories, but it is not so straightforward as competitive levels are higher. Further, it is hypothesized that players who have greater physical attributes may have more chances to be selected for competition and consequently, more minutes of playing (MP). Also, operating growth hormones may have a significant role on playing time, as it is associated with maturation levels and therefore, to greater physical qualities [29–31].

Short-term maturity status, and physical-based approach of selection for competition and time of play may have a double negative impact on elite young player’s careers. In fact, the non-protection of later maturing players may lead to dropouts and lesser time of play, and in the case of early maturing players, not reaching a senior contract [32, 33]. However, there is still a lack of evidence [26], as far as we know, supporting the impact of a multifactorial perspective on the selection for competition and time of play. In fact, this multifactorial approach including the maturity status, level of fitness, and the role of hormonal changes influencing the selection and time of play should be considered as it may interfere with coaches’ choices and practices. This approach would give a more robust information considering the current determinants influencing the selection and time of play strategies of academy soccer clubs.

For these reasons, the purposes of this study were: (i) to analyze the correlations between MP with the skeletal age, maturity offset, maximal oxygen uptake (VO_2max_), fatigue index, countermovement jump (CMJ) performance, GH, and IGF1 levels; and (ii) to show a regression analysis that explain the MP in each competitive level with the above independent variables (i.e., maturation status, physical fitness and hormonal levels).

## Method

### Experimental approach to the problem

This research was conducted as a prospective study with observational cohort design which was performed on a cross-sectional basis, yielding practical results. Researchers have checked players over the whole season and assessments were performed upon completion of the competitive season. The outline of the research can be seen in Table 1. All assessments were done after the monitoring during the season and the same weather conditions (temperature 21–23 ° C and humidity 50%) after the end of the season. After three days of recovery from the last training players were assessed by anthropometric, hormonal, and imaging of left-hand radiography in day 1, the CMJ was evaluated on day 2, the seven repeated sprint tests (7RST) test and the Yo-Yo Intermittent Recovery Test level 1 (YYIRT1) were tested in the last two days, respectively. All tests were performed in the morning [34].

**Table 1:** During control in season and assessment

Year	2018								2019			
Month	July	Aug		Sept	Oct	Nov	Dec	Jan	Feb	Mar	End of Mar	Total
Phase	First PP (6 week)		Provincial Games						Second PP (6 week)	NG	Assessment	
OG			2	4	4	4	4	4		4	4 days	26
NOG		2							4			6

* *OG* Official Games, *NOG* Non-Official Games, *PP* Preparation phase, *NG* National Games

### Subjects

Sample in this study were twenty-six elite youth football player (Mean ± Standard deviation; chronological age: 14.6 ± 0.2 years; height: 172.4 ± 7.5 cm; body mass: 59.4 ± 10.1 kg; skeletal age: 15.0 ± 1.0 years; maturity offset: 1.1 ± 0.5 years; VO_2max_, 48.7 ± 2.9 ml.kg^−1^.min^−1^). The age group of these subjects was U15 which they played in the highest level of their age group, according to the program of the relevant federation, they first participated in the provincial league and then in the national league. Among the player 3 were goalkeepers, 11 defenders, 4 central midfielders, 4 wingers and 4 attackers. The inclusion criteria were: 1) at least 3 years playing soccer background; 2) Active and regular participation in all stages of the study; 3) they did not allow to use any supplement that effect on growth and maturation; and 4) participants were not permitted to perform additional exercises. The exclusion criteria: 1) not participating in 80% of competitions (formal and no formal) and training sessions during the season; 2) did not attend in one of the medical or physical tests of the study. If any players who competed for a limited time during the match each week. Then we provided a friendly match or a small side game. This study was authorized by the Ethics Committee of the University of Isfahan (IR.UI.REC.1399.001). Also we have done it regarding the Helsinki declaration (2013). All the participants were informed about the risks and benefits of this study, have the right to quit in each part of that they want. Informed consent was obtained from the parents/ young players for the participation of the study. To measure the YYIRT1 and 7RST tests, players wore stock shoes. However, players for CMJ used running shoes.

### Procedures

#### Anthropometric measurements

For anthropometric measurement, we assessed three variables (weight, standing height, and sitting height) and all measurements were carried out in the morning [35]. SECA audiometer 2013, “Hamburg, Germany” was utilized to measure standing height and sitting height. As with previous studies considerations [36] participants, stood as near as possible to the stadiometer with bare feet, and their head, back, and shoulder should attach to the stadiometer with feet positioned beside every other to assessing standing height. For sitting height, they sat on the 50 cm box with buttocks as close as possible to the stadiometer and their posture should be upright. The difference between the stadiometer number and the box height will be the sitting height, also for estimating maturity offset we needed leg length which is calculated by differences between standing, and sitting height. The last assessment in this part was the weight that was evaluated by SECA, model 813, England with an accuracy of ±0.1 kg. To estimate maturity offset we used Mirwald equation which is: Maturity offset = −9.236 + 0.0002708 (leg length × sitting height) - 0.001663 (age × leg length) + 0.007216 (age × sitting height) + 0.02292 (Weight by Height ratio), R = 0.94, R2 = 0.891, and SEE = 0.592) and for leg length = Standing Height (cm) - Sitting height (cm) [4].

#### Skeletal age

Skeletal age is the most acceptable method for assessment of maturity level. 2D X-ray radiographs of the hand and wrist used for verifying skeletal age regarding this purpose we used the EOS imaging system to specify skeletal age. The EOS imaging system is a new device that can provide lower radiation dose 50 to 85% [37, 38] than digital radiography as well as better image quality [39, 40]. There is some technique for assessing the skeletal age but the most reliable is the Fels method [6], which is we used in this study by our expert. In the Fels method assessor use some criteria that relate to the level of maturity and ratio of hand and wrist bones also we need chronological age that is differences between the date of assessment and birth date. After identifying the ratio and maturity levels of bones, data, and chronological age inserted to the Felshw 1.0 software to evaluated the skeletal age. For identifying the maturity status of the player, we had to subtract the skeletal age from the chronological age then if this number was more than +1 the player identifies as early, if this subtract was less than −1 he identifies as late and if it was between +1 and − 1 he was average mature.

#### Blood sample analysis

The same previous studies [31, 41, 42], after at least 12 h of fasting and 72 h after the last training, players went to the Al-Zahra Hospital laboratory for taking 10 ml blood. Blood was drawn from the antecubital fossa, this blood was drawn at 8 a.m. and the samples were quickly centrifuged. To quantify GH and IGF-1 levels we used the serum obtained from the blood. In this, part Chemiluminescence technique (ICMA) and IMMULITE framework (2000xpi Systems, organization SIEMENS Germany) were utilized. The sensibility of the kit that was used for GH (REF: L2KGRH2; lot 171) was 0.01 ng/ml and for IGF-1 (REF: L2KGF2 and lot: 571) was 13.3 ng/ml.

#### Countermovement jump test

The CMJ test was used to investigate explosive lower-body power [43]. Players warm up about 15 min, they starting with running slowly after that 5 × 10 m running with maximum speed, horizontal and vertical hop, and CMJ drills. Players before doing CMJs carried out 2 trials for familiarizing. The first test started when the examiner said jump and player should have stood on the mat with 90-degree flexion in the knee then they jump vertically maximum energy. Players stood on the electronic pad during the CMJ test with their hands stable, without swinging, on his lateral area of the pelvic. The second test did after 5 min recovery, finally, the best performance was recorded in cm [44].

#### Repeated sprint test

The 7 RST is one of the best reliable tests regarding similarity with the football and this test assess anaerobic power. Participants should run in maximum speed one by one until the 7th run, and between each curved run, the players had 25 s recovery for came back to the start point. Players should warm up 15 min by jogging, stretching, and sprints. For the test, the player was standing in the start line then started his performance as fast as possible by command of the assessor until the finish line, then he ran back to the start line as for the limited time (25 s). The fatigue index was calculated by subtraction of the worst and the best performance [45]. If participants lost one of their runs, an average of 6 other runs registered as the failure test. Newtest Power timer 300-series testing system (Newtest, made in Finland) which has photo finish sensors and jump mat used to monitor this test and CMJ test. Photo finish sensors were located at the start and finish line in this test.

#### Yo-Yo intermittent recovery test

The YYIRT1 was the last test performed and it was used to estimate the VO_2max_. Standard warm-up was done by a conditioning coach and the procedure was like the 7RST test. In the YYIRT1 participant run 40 m back and forth then they had 10 m back and forth recovery. The test is conducted at 10 km/h and increase 0.5 km/h for the next step. The end of this test happened when each of the participants had two failures for being in the line at the same time with the beep sound, then the level which player could not catch register as the record. VO_2max_ evaluated by this equation: VO_2max_ (ml.kg^-1.^min^−1^) = IR1 distance (m) × 0.0084 + 36.4 [46].

### Statistical analyses

For statistical analysis, first, the normality of the information was checked with Shapiro-Wilk, then the mean and standard deviation (SD) were used to describe the study samples. Pearson and Spearman’s correlations were used for normal and non-normal data, respectively. Considering the following correlation thresholds [47]: < 0.1 = trivial; 0.1–0.3 = small; 0.4–0.5 = moderate; 0.6–0.7 = large; 0.8–0.9 = very large; and > 0.9 = nearly perfect. Ultimately, regression analysis was performed in two stages. First, for all dependent information, linear regression was performed with MP and obtained predicted formula and residual them, and in the next step, multiple linear regression was performed between dependent and independent information. All statistical analyses were done to use GraphPad Prism version 8.0.1 with considering the significance surface was at P < 0.05.

## Results

Descriptive characteristics of players are presented in Table 2. Values are reported as mean ± SD. Total season were 1411 ± 242.6 MP in matches and 32 matches were during the study period.

**Table 2:** Descriptive characteristics of players

Variables	Mean ± SD
Height (cm)	172.4 ± 7.5
Weight (kg)	59.4 ± 10.1
Chronological age (years)	14.6 ± 0.2
Skeletal age (years)	15.0 ± 1.0
Maturity Offsets (years)	1.1 ± 0.5
Soccer training (months)	80.3 ± 23.0
VO_2max_ (ml.kg^−1^.min^−1^)	48.7 ± 2.9
Fatigue index (seconds)	0.6 ± 0.3
Best sprints (seconds)	6.6 ± 0.4
Worst sprints (seconds)	7.1 ± 0.3
CMJ (cm)	39.5 ± 5.98
GH (ng/dl)	1.9 ± 1.4
IGF1 (ng/dl)	461.5 ± 110.6
Minutes of playing (min)	1411 ± 242.6

* *VO*_*2max*_ maximal oxygen consumption, *CMJ* countermovement jump, *GH* growth hormone, *IGF1* insulin-like growth factor

**Fig. 1:**
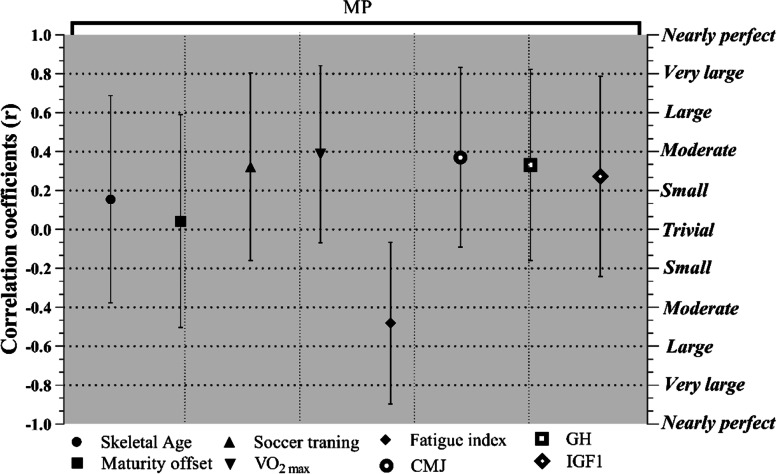
Correlation coefficients (95%CI) the minutes of playing soccer player in the compositions with the skeletal age (years); maturity offset (years); Soccer training (months); VO_2max_ (ml.kg^−1^.min^−1^); Fatigue index (seconds); CMJ indicates the best of countermovement jumps performance (cm); GH, and IGF1 levels (ng/dl)

Figure 1 shows the correlations between MP with maturation status, fitness status and hormonal levels, with correlation coefficients of CI 95%. Based on the result of this study, there was a significant moderate correlation between CMJ (*r* = 0.42; CI 95% [0.04 to 0.69]; *p* = 0.03) and VO_2max_ (*r* = 0.44; CI 95% [0.06 to 0.71]; *p* = 0.02) and MP.

Hence, fatigue index (*r* = −0.54; CI 95% [−0.66 to 0.02]; p ≤ 0.004) was largely related to MP. VO_2max_ was largely correlated to CMJ (*r* = 0.51; CI 95% [0.16 to 0.75]; *p* = 0.007) and GH (*r* = 0.57; CI 95% [0.22 to 0.79]; *p* = 0.003), and also a large correlation was observed between GH and CMJ (*r* = 0.55; CI 95% [0.20 to 0.78]; *p* = 0.003). The relationship between fatigue index and soccer training (*r* = −0.41; CI 95% [−0.69 to −0.01]; *p* = 0.039) was shown to be moderate (Table 3).

**Table 3:** Pearson and spearman correlation analysis

Variables	β0	β1	β2	β3	β4	β5	β6	β7	β8
MP (β0)	1								
Skeletal Age (β1)	0.18	1							
Maturity offset (β2)	0.05	0.66	1						
Soccer training (β3)	0.37	0.04	0.15	1					
VO_2max_ (β4)	**0.44**	−0.01	−0.08	0.10	1				
Fatigue index (β5)	**−0.54**	0.03	0.24	−0.41	−0.20	1			
CMJ (β6)	**0.42**	0.37	0.36	−0.05	**0.51**	0.18	1		
GH Level (β7)	0.38	0.03	−0.11	0.01	**0.57**	−0.09	**0.55**	1	
IGF1 Level (β8)	0.32	−0.12	0.08	0.04	0.27	−0.02	0.24	0.31	1

* *MP* minutes of playing, *CMJ* countermovement jumps, *VO*_*2max*_ maximal oxygen consumption, *GH* growth hormone, *IGF1* insulin-like growth factor. Significant differences (*p* ≤ 0.05) are highlighted in bold

**Fig. 2:**
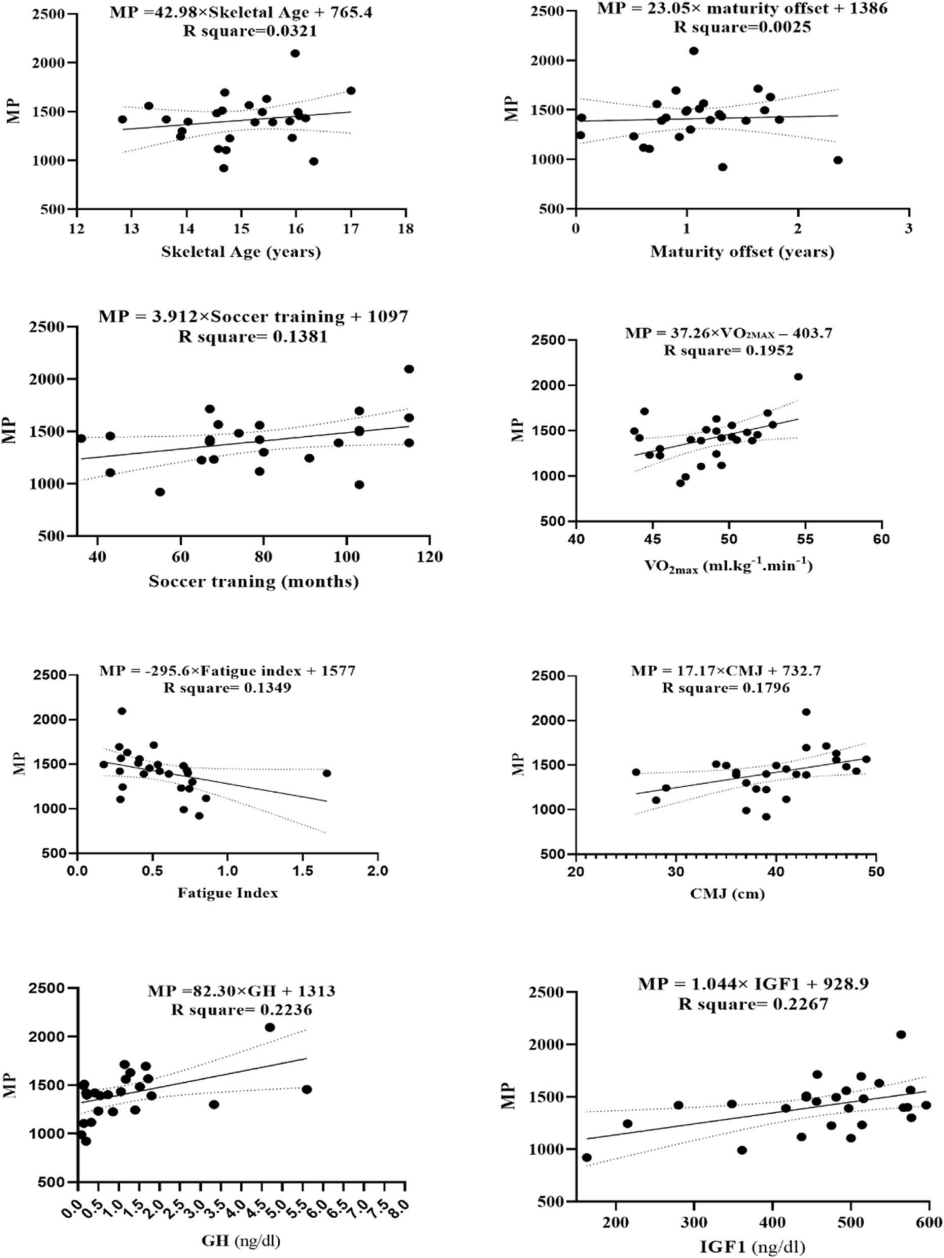
Regression analysis to explain MP (the minutes playing in each competitive level). VO_2max_ = maximal oxygen consumption; CMJ = countermovement jump; GH = growth hormone; IGF1 = insulin-like growth factor

**Fig. 3:**
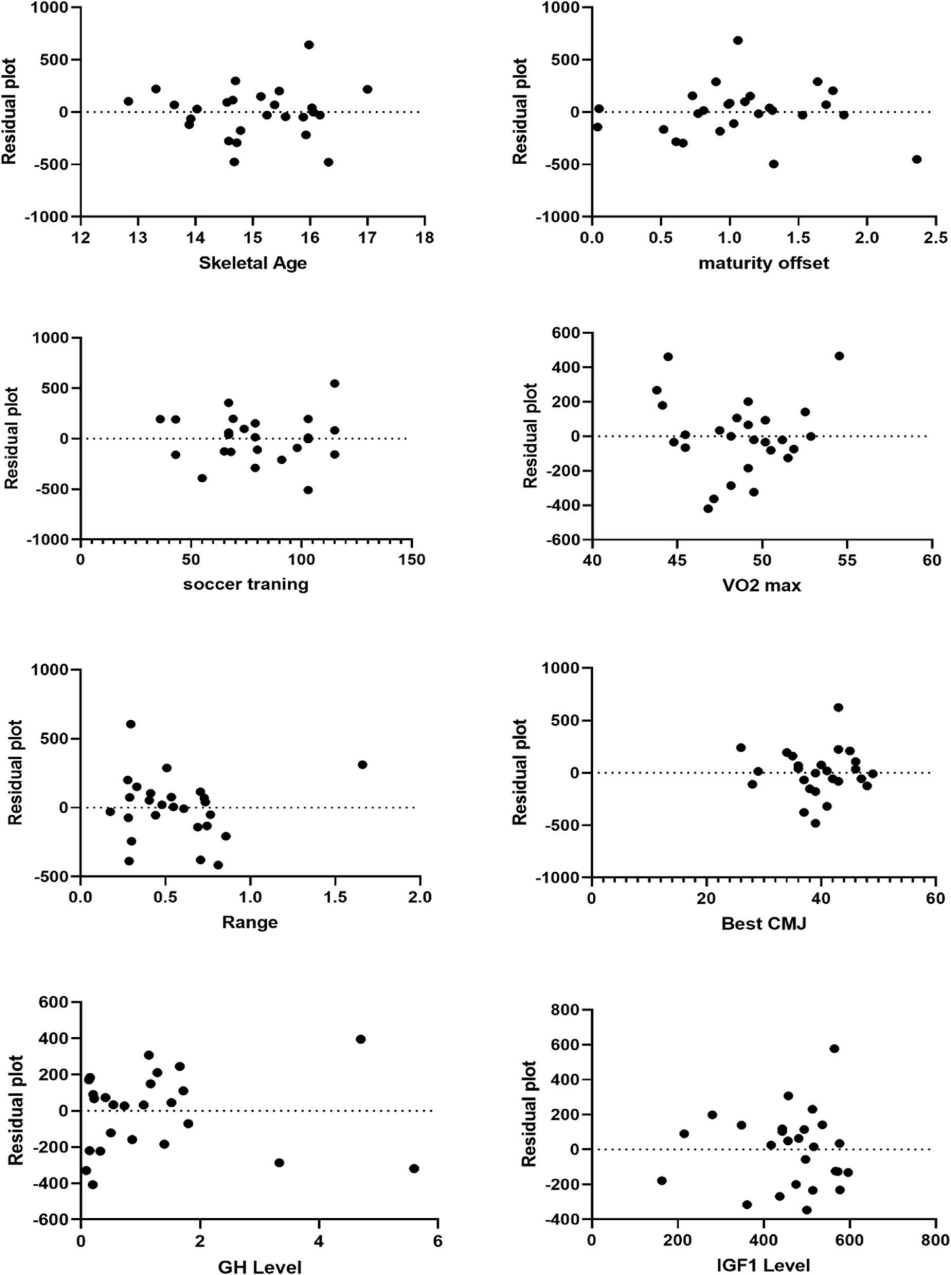
Residual plot; the difference between the actual value of the dependent variable and the value predicted by the residual provided. MP = minutes of playing; VO_2max_ = maximal oxygen consumption; CMJ = countermovement jump; GH = growth hormone; IGF1 = insulin-like growth factor

The MP and independent variables (i.e., maturation status, fitness status, and hormonal levels) with linear regression are reported in the diagram, respectively (Fig. 2). The results of this study showed that MP significantly predicted the levels of VO_2max_ (F (1, 24) = 5.82, b = 37.26, *p* = 0.024), with an R^2^ = 0.20; CMJ (F (1, 24) = 5.26, b = 17.17; *p* = 0.031) with an R^2^ = 0.18; GH (F (1, 24) = 6.91, b = 82.30; *p* = 0.015) with an R^2^ = 0.224, and IGF1 levels (F (1, 24) = 7.04, b = 1.04; *p* = 0.014, R^2^ = 0.23. Participants predicted the competitive level (MP) increasing 37.26 min for each ml.kg^−1^.min^−1^ of VO_2max_; 17.17 min for each cm of CMJ; 82.30 min for each ng/dl of GH and increased 1.02 min for each ng/dl of IGF1. And ultimately for find out better predictions, the residual plots were also showed in Fig. 3.

Multiple linear regression analysis was calculated to predict MP based on maturation status, fitness status, and hormonal levels (Table 3). A significant value was found (F (8, 17) = 3.47, *p* = 0.015), with an R^2^ of 0.62. Participants predicted MP (Y) is equal to Beta0 + Beta1 (Skeletal age) - Beta2 (Maturity offset) + Beta3 (Soccer training) - Beta4 (VO_2max_) - Beta5 (Fatigue index) + Beta6 (CMJ) + Beta7 (GH) + Beta8 (IGF1), where maturation status, is evaluated as years, fitness status as months, and ml.kg^−1^.min^−1^, seconds, and cm based on the equation, respectively. However, none of the variables were able to predict the competitive level (MP) of the soccer player U15 individually (Table 4).

**Table 4:** Multiple linear regression analysis: minutes of playing with all variables

Variables	Beta	Estimate	|t|	P	95% CI for estimated
MP (min)	β0	309.2	0.28	0.78	[−2024 to 2642]
Skeletal Age (years)	β1	14.5	0.28	0.79	[−96.4 to 125.4]
Maturity offset (years)	β2	−75.9	0.72	0.48	[−297.8 to 145.9]
Soccer training (months)	β3	3.2	1.75	0.09	[−0.6 to 6.9]
VO_2 max_ (ml.kg^−1^.min^−1^)	β4	−1.8	0.01	0.92	[−39.2 to 35.7]
Fatigue index (seconds)	β5	−219.4	1.53	0.15	[−521.9 to 83.2]
CMJ (cm)	β6	14.1	1.61	0.13	[−4.3 to 32.5]
GH (ng/dl)	β7	44.3	1.39	0.18	[−22.9 to 111.5]
IGF1 (ng/dl)	β8	0.7	1.87	0.08	[−0.1 to 1.5]
				**R**^**2**^ = 0.62	

* *CI* confidence interval, *MP* minutes of playing, *VO*_*2max*_ maximal oxygen consumption, *CMJ* countermovement jump, *GH* growth hormone, *IGF1* insulin-like growth factor. Significant differences (*p* ≤ 0.05) are highlighted in bold

## Discussion

The purpose of the present study was to analyze the relationships between MP with maturity status, fitness, and hormonal levels and to explain how those measures may influence the MP. The main findings of the present study were that the MP were positively correlated with VO_2max_ and CMJ in a moderate magnitude, and negatively correlated with fatigue index, with a large magnitude. Moreover, VO_2max_ had large relationships with growth hormones (GH and IGF1) and CMJ. Also, growth hormones were largely related with CMJ. The model of multiple linear regression using maturation status, fitness status, and hormonal levels explained 62% of the MP. However, isolated independent variables were not able to determine the MP by the model.

The first aim of the present study was to analyze the correlations between MP and maturation levels (including hormones) and physical performance. Our results showed no correlation between maturation and MP, maybe because our sample did not differ significantly neither on skeletal age, nor in maturity offset. Moreover, all the sample have already the maturity offset at least half a year ago. The lack of correlations between skeletal age, maturity offset, MP, VO_2max_ and fatigue index, may also suggest that biological maturation was not considered by the related stakeholders for selecting players for match participation. Thus, skeletal age may be independent of hormonal activity and development, although this remains unclear [48].

Other studies tested the correlations between maturation status and physical performance [49], showing that early maturing boys tend to be more successful in soccer in mid- and late adolescence. In opposition, Cunha et al. [50] showed that biological maturation does not affect neither ventilatory thresholds, nor VO_2max_ values of young soccer players. However, different maturity evaluation was conducted in those different studies, suggesting that more studies should be conducted in this field. Nevertheless, for general adolescent male population, data suggest that strength and power attain maximal growth after maturity offset, running speed, and maximal aerobic power [12].

The correlation between MP and fitness status was also included in the analysis, being registered moderate correlations between VO_2max_, CMJ and MP. In fact, a significant correlation between aerobic capacity and distance covered during a match have already been found [51]. It was suggested that an enhancement of 6% in VO_2max_ can improve soccer performance and more specifically can increase the distance covered, the number of sprints, and the number of actions with the ball, in male elite soccer players [52]. Moreover, Murtagh et al. (2018), noticed that, among others, vertical CMJ was an indicator of elite youth soccer players. Those findings suggest that players with better physical performance may have more chances to be selected, and consequently, have more MP.

The relationships between maturation status and physical performance, and the relationships between hormonal activity and physical performance has been already analyzed in other studies [17, 53, 54]. The increase of GH in plasma after exercise has been well documented [55]. According to the literature, the duration and intensity of training and its type have a significant impact on IGF-1 concentration [56], with the GH / IGF-I actions influencing fuel compound metabolism such as protein metabolism and body composition [57]. Our results revealed a large correlation between GH and VO_2max_, and between GH and CMJ. In fact, a study conducted on 18 elite soccer players from U17 competitive level revealed that pubertal growth combined with soccer training increased the concentrations of GH and that higher levels of IGF1 were correlated with better jump performance [17]. For those reasons, coaches should consider not only the pubertal growth in isolation, but also, its complementation with the imposed soccer training dose that promote greater hormonal activity. Indeed, a longitudinal study examining the relationship between maturity status and physical performance revealed that as children were closer to PHV they presented greater jump performance as well as an increased maximal oxygen uptake and anaerobic capacity, which is in concordance with the sample of this study [49].

Considering the influence of the independent variables analyzed on the MP, a linear regression was executed for each variable. Results revealed that the MP were influenced by 23% by IGF1, 22% by GH, 20% by VO_2max_, and 19% by CMJ. Interestingly, a study comparing the physical performance between academy and non-academy players revealed that long-term soccer training programs are associated with better physical performance, independently of levels of maturation [58]. In that study, the authors stated that the rate of performance development must consider the maturation influence. Notwithstanding, our results seems to be somewhat predictable, as children of our sample age are expected to be at a critical stage of growth, where circulating hormones become increasingly active [17, 21]. Indeed, this internal anabolic environment facilitates growth and consequently, maturation levels [20]. Also, given the fact that at the U15 competitive level there are differences in biological ages among players of the same team, the more advanced players in maturity are expected to have greater levels of physical performance, and therefore have greater chances to be selected [32]. From these evidences, the operating hormones together with physical qualities appear to be preponderant in the selection for competition and in the MP and must be accounted for coaches in the process of talent selection and development.

Although simple linear regressions may establish a relationship between the response (dependent) and explanatory (independent) variables, it is difficult that a single independent variable predicts the true value of the dependent variable [59]. This is due to the fact that other dimensions may influence the response of the actual dependent variable. After running the multiple linear regression (multifactorial approach), our results revealed that maturity status, hormonal and fitness levels interactions determined 62% of the MP. This fact may be responsible for the bias in the selection of players based on the current physical state in young athletes, instead of being based on the technical/tactical potential. Thus, this can potentially lead to the dropout of the later maturing players, causing negative consequences for talent selection processes [3]. Despite that, when analyzing the independent variables alone (in the current model) it was not possible to significantly determine the MP. There are few evidences supporting what influences the MP [26, 32], however, Deprez et al. [32] through a multiple linear regression model revealed that explosive power was the most determinant factor explaining 16.7% of the variance in future MP. Moreover, as mentioned earlier, our model was not able to assign influence on the MP to any of the explanatory variables when analyzed individually. In fact, better physical attributes are considered a determinant factor for selecting players for competition, being this associated with early maturing players as they tend to present greater physical qualities due to their advanced maturity status [3]. Furthermore, it seems unwise to pay attention only to independent variables alone, as a multifactorial approach (interacting explanatory variables) may give a more robust perception of the influencing factors on MP.

This study presented some limitations. One of the major limitations is related to the sample size in which only one U15 team was analyzed. Also, other contextual dimensions such as the magnitude of training (volume, intensity, frequency) were not considered. Given that, longitudinal studies using greater sample sizes and incorporating a multifactorial approach, as well as possible positional dependencies influencing selection for competition and MP should be considered. Another limitation of the present study is the lack of evaluations in the pre-season and mid-season, which could not be done in this study. We strongly encourage researchers to consider this in future studies. Also, given the fact that in our sample age-group a 2-year variation can be considered a critical aspect at maturation level, future studies should categorize the sample into two groups based on their skeletal age to compare the between-group differences. Nevertheless, to the best of our knowledge, this was the first study analyzing the influence of maturity, hormonal activity, and physical variables on the selection for competition and MP. As the interaction of these variables explained a great percentage of the MP and considering the relationships between growth hormones and physical performance, and MP, coaches should consider these dimensions when selecting players and determining the MP. Also, it may decrease the risks that are associated with unidimensional-based approaches.

Considering the relevance of hormonal production and maturation on physical performance and match play, possibly coaches should be aware of that to minimize the impact of that on player’s selection. In fact, maturation follows his own rhythm in different players, and a selection exclusively based on momentary physical performance may create conditions for bias in the process. Eventually, additional criteria as declarative and processual tactical knowledge and technical potential should be added as determinant criteria in selection, aiming to avoid that later mature players may be excluded by physical performance possession similar or better tactical/technical conditions. Multidimensional levels are important to employ future selection criteria.

## Conclusions

Summarizing, large correlations between growth hormones and physical performance were found. Also, CMJ and VO_2max_ were correlated with the time played in the matches. Moreover, despite the model was not able to able to attribute influence of isolated independent variables to MP, when using the multifactorial approach, the model explained 62% of the MP Considering the current evidence and the study limitations it seems that growth hormones activity and physical fitness have a determinant impact on the selection for competition and MP.

## Data Availability

The datasets generated and analysed during the current study are not publicly available due to ethical restrictions, however, they are available from the corresponding author on reasonable request.
